# Robotic repair of the symptomatic vesicouterine fistula

**DOI:** 10.1007/s00192-020-04558-0

**Published:** 2020-10-20

**Authors:** I. E. Govorov, M. A. Vorobeva, E. V. Komlichenko

**Affiliations:** 1 Almazov National Medical Research Centre, Institute of Perinatology and Pediatrics, 197341, Akkuratova str.2, Saint-Petersburg, Russia; 2 Women’s and Children’s Health Department, Karolinska Institutet, 17176, Solnavagen 1, Solna, Sweden; 3 Regional Perinatal Centre, Kursk, Russia

**Keywords:** Vesicouterine fistula, Urogynecology, Robotic surgery

**Fig. 1:**
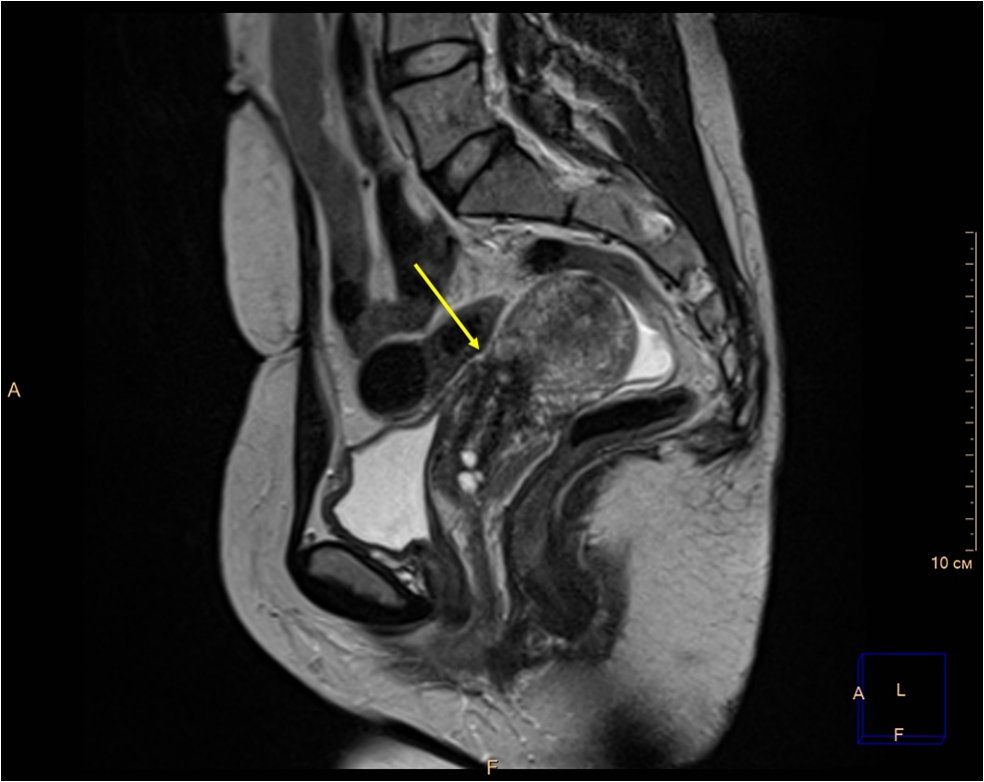
Pelvic MRI, T2-weighted sagittal plane. The *yellow arrow* indicates the cesarean section scar. No signs of a vesicouterine fistula are present

**Fig. 2:**
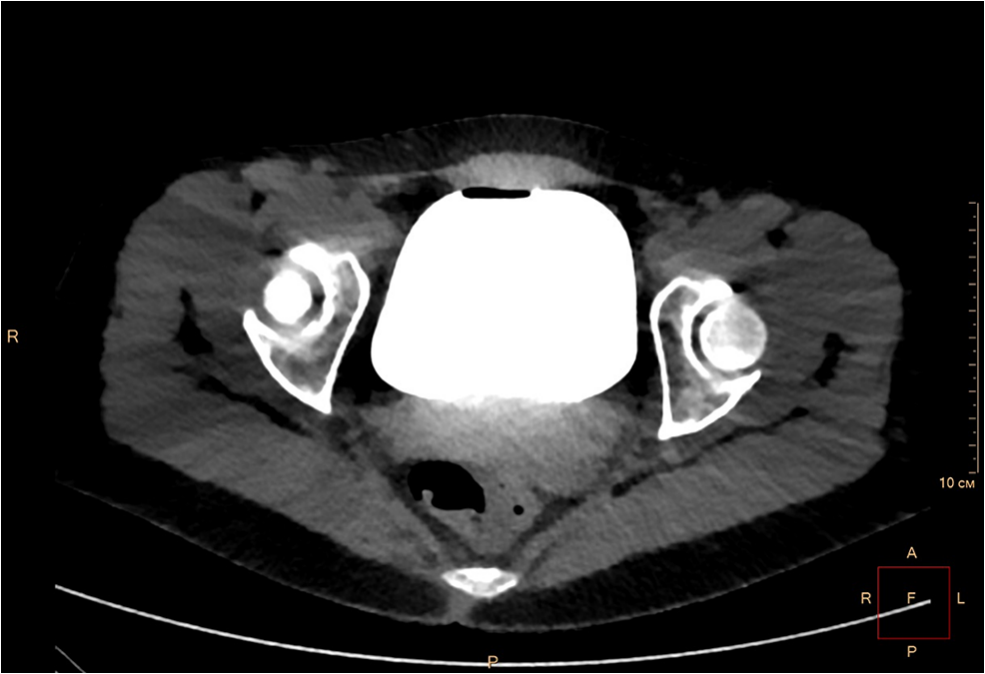
CT cystography, axial plane (retrograde contrast medium instillation). Contrast medium accumulated within the bladder without leakage

**Fig. 3:**
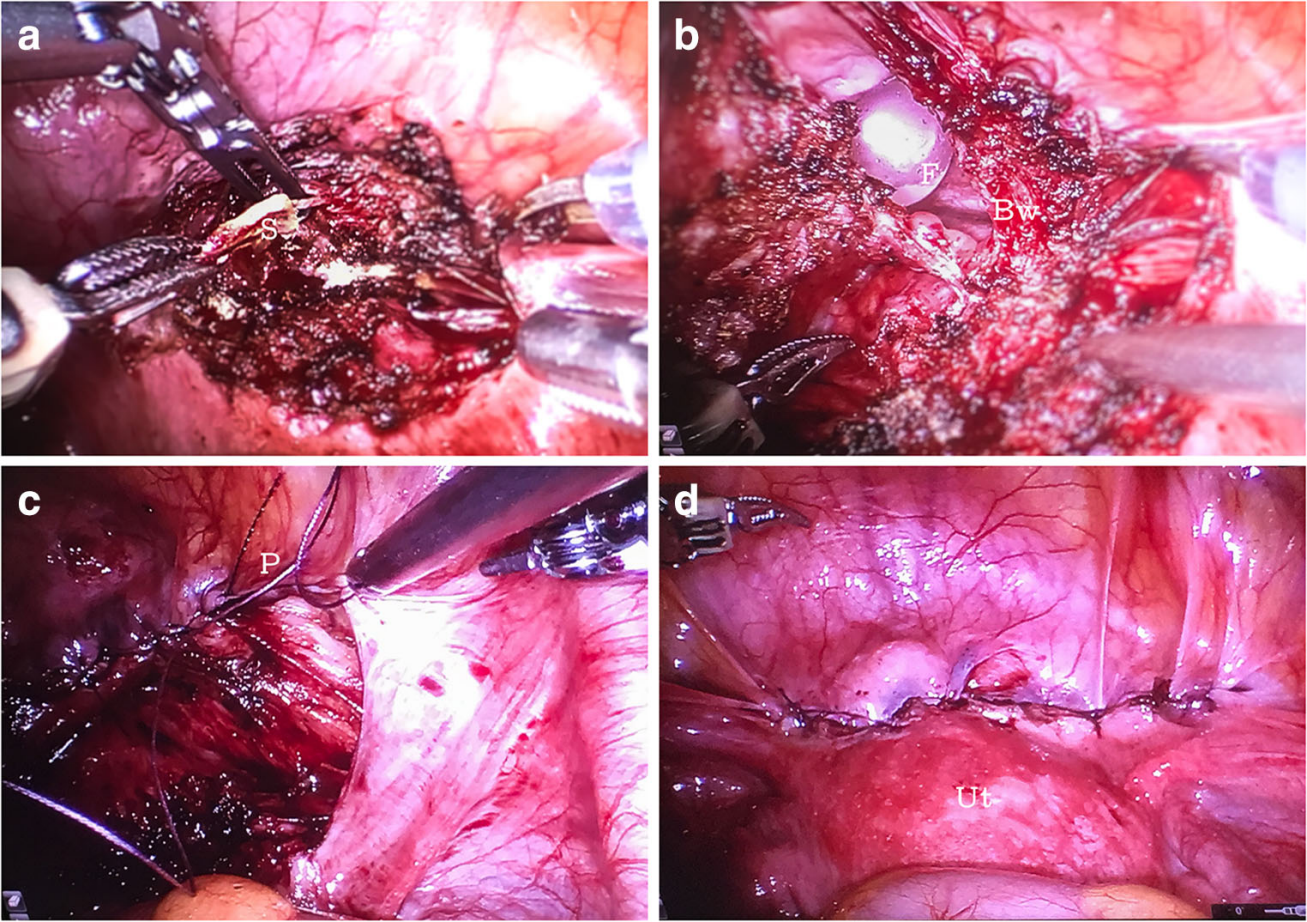
**a** Removing the remnant suture (*S*) within the scar tissue. **b** Fistula excision and repair. Foley catheter (*F*) in the bladder (*Bw*). **c** Peritonization. *P* peritoneum

Vesicouterine fistula (VUF) is an abnormal connection between the uterine cavity and bladder, most commonly formed following cesarean section (CS) [1]. VUF accounts for up to 4% of all genitourinary fistulas and manifests with various degrees of urinary incontinence, cyclic hematuria, and amenorrhea [2, 3]. Here we describe a 32-year-old woman who presented with gross hematuria and urinary incontinence, both cyclical, starting 2 days before and continuing along with the menstruation. The patient previously had two uneventful CS, 3 years apart. Following the admission, the patient underwent repeated cystoscopies that failed to discover the cause. Bladder endometriosis and fistula were considered to be the main competing conditions. Repeated cystoscopies found neither endometriotic lesions nor signs of a fistula. Contrast MRI revealed that the anterior wall of the uterus in the area typical for CS was thinned, whereas the bladder wall was pulled up to the postoperative scar (Fig. 1). However, there was no leakage of contrast medium. The patient underwent CT cystography with retrograde instillation of contrast medium, which did not bring any new facts as no contrast medium leakage was detected, probably because of the underfilled bladder (Fig. 2). In order to exclude bladder malignancy, the patient was consulted by the oncologist and underwent another cystoscopy. The latter revealed two pinhole mucosal retractions, 3 cm above the orifice of the right ureter, with a small amount of blood coming through them. The patient was scheduled for robotic fistula repair. The surgery took 90 min and included removal of the remnant suture (~1 cm long), fistula excision, and repair of the bladder wall and uterine scar defect (Fig. 3). The patient was discharged on day 5 and has remained asymptomatic so far. We believe that the robotic approach is optimal for repairing VUF, taking into account the size of the fistulas and the probability of the disturbed anatomy due to previous CS.
